# Abuse, Toxicology and the Resurgence of Propylhexedrine: A Case Report and Review of Literature

**DOI:** 10.7759/cureus.10868

**Published:** 2020-10-09

**Authors:** Nikhil Teja, Caroline P Dodge, Cornel N Stanciu

**Affiliations:** 1 Psychiatry, Dartmouth-Hitchcock Medical Center, Lebanon, USA; 2 Psychiatry, Geisel School of Medicine, Concord, USA

**Keywords:** propylhexedrine, benzedrex, parachuting, stimulant abuse

## Abstract

Propylhexedrine, the active ingredient in over-the-counter nasal decongestants, carries significant abuse potential for users seeking psychostimulant effects. Historically, propylhexedrine was perceived to have a good safety profile resulting in endorsement of it replacing the highly abused amphetamine sulfate as the active ingredient in nasal decongestants in 1949. While much of the published literature concerning its psychoactive potential comes from the 1970s and 1980s, we have encountered several recent cases of toxidrome secondary to its abuse. Awareness of the hazards associated with this pharmaceutical should be of interest to physicians of all specialties who are likely to encounter such cases, as well and legislators interested in exerting regulatory control. Here we review all existing literature concerning this pharmaceutical compound.

## Introduction

Propylhexedrine is widely used medicinally for the relief of nasal congestion due to colds, allergic rhinitis and sinusitis under the trade name Benzedrex® [1]. With each label dose instruction of two inhalations per nostril every two hours, it delivers 0.4 to 0.5 mg of propylhexedrine. In Europe, propylhexedrine has also been marketed as the anorectic ingredient in weight loss agent (Obesin). For this indication, the approved doses range from 5 to 30 mg [2]. Propylhexedrine was initially developed as a substitute for amphetamines in the 1940s in response to abuse and deaths from amphetamines which were extracted from the inhalers. Unlike amphetamines, propylhexedrine is not on the US schedule, yet it is a structural analogue of amphetamine. Recreationally, in doses of 100 to 300 mg orally, propylhexedrine has been explored for its psychostimulating properties as a “legal high”. This was first reported in the literature in 1970 in New Zealand in a case reporting acute psychosis reminiscent of amphetamine psychosis [3]. Numerous case reports emerged in the American literature in the 1970s and 1980s, and while there was a relative surge in cocaine and methamphetamine use during this period, Smith et al. did not find an increase in its abuse during this period after studying surveying data from several major metropolitan areas [1]. In the 1990s and 2000s there was a single published case report involving death from intravenous injection. Propylhexedrine toxicity reports returned to the literature in 2011 with a fatality involving its combination with Mitragynine, an active constituent of Kratom [4]. There is no data regarding the prevalence of current propylhexedrine abuse, and national surveys do not track it.

## Case presentation

A 25-year-old male with history of stimulant use disorder was admitted to an inpatient psychiatric facility for an acute episode of agitation and disorganized behaviours to the degree of assaulting family members. Prior to admission, a thorough workup was done, and urine toxicology was unremarkable while laboratories were unrevealing of any etiology. The patient had been attending an Intensive Outpatient Program (IOP) geared towards addressing his substance use for the past few weeks and although his behaviour was erratic at times in the days leading up to admission, his urine toxicology was negative for all tested illicit substances.

On evaluation the day following admission, the patient appeared euthymic and denied all psychiatric review of symptoms elements. He disclosed struggling with sobriety from stimulants and feeling “trapped” by having to participate in the court mandated the IOP and having to comply with negative urine screens.

Patient relates a past diagnosis of ADHD at age 16 and past treatment trials with methylphenidate and mixed amphetamine salt compounds. A few years later he was introduced to methamphetamine by a friend and discovered a more intense effect, allowing him to stay up for several days, concentrate, and have stamina. He would insufflate but also smoke it and at times use has resulted in some confusional states, abnormal perceptions and “white outs” where he would commit actions without remembering. One of these actions include a motor vehicle accident he was involved in while intoxicated and as a result he was court mandated to complete the IOP. Over the past few years he has not been able to stay away from methamphetamines and endorses extreme cravings when not available and “crashing” when coming off a few days of binging. Playing video games and engaging in sexual activity always elicits extreme cravings.

Several weeks prior to presentation he read on the dark web about propylhexedrine, a methamphetamine-like compound which some users reassured would not show up on a standard drug screen. He decided to “give it a try” and began purchasing nasal decongestant inhalers from the local convenience store, taking the cotton filaments out which are soaked with the compound, and swallowing three of them at a time. Most recently he has been “parachuting” these by wrapping the cotton filaments in tissue paper prior to swallowing. This way he noted a more intense yet sustained feeling once the tissue gradually dissolves in the gastrointestinal tract and exposes the cotton filaments for absorption. He last ingested this the morning of admission to the hospital. He described the euphoric high from propylhexedrine as an intermediate between Adderall and methamphetamine.

## Discussion

Methodology

We searched databases for English literature published between 1975 and 2020 using predefined keywords such as “propylhexedrine”, “benzedrex”, “methamphetamine analogue AND abuse”, “decongestant AND abuse”. All human literature and select animal studies were considered for inclusion. We supplemented with citations gleaned from search returns as well as other sources. All levels of evidence were considered for review.

Pharmacology

On ingestion, propylhexedrine is rapidly absorbed from the gastrointestinal tract [2]. In Maurine intravenous propylhexedrine trials, maximum concentration in the central nervous system (CNS) paralleled plasma concentrations with a 10:1 brain:plasma ratio [2]. In humans, the toxic effect of propylhexedrine when abused is the result of a relatively greater efficacy in peripheral adrenergic stimulation compared to the sought-after CNS effects [5]. Some report one-twelfth of the CNS stimulant effect and one-eighth of the vasopressor effects of amphetamines [6].

Structurally similar to amphetamine, propylhexedrine found in nasal decongestant inhalers is racemic, with the laevorotary isomer acting as the predominant releaser of norepinephrine and dopamine in the CNS [7]. Propylhexedrine mimics the actions of norepinephrine and epinephrine by antagonising alpha and beta-adrenergic receptors in the mucosa of the respiratory tract. Propylhexedrine reverses the direction of flow of norepinephrine, dopamine and serotonin (5HT) transporter leading to release of these neurotransmitters from vesicles into the cytoplasm, and then from the cytoplasm into the synapse [8,9]. Further release of neurotransmitters is facilitated by antagonism of VMAT2.

Methods of use and behavioural pharmacology

Nasal decongestant inhalers are the most common source of propylhexedrine. Users forcefully open the encasing to obtain the cotton-containing propylhexedrine. The cotton swab can then be cut into pieces and swallowed, or placed in a liquid (typically with acidic properties such as soda or lemon juice) for an extended period to extract the propylhexedrine [5,10]. The extracted propylhexedrine can be “parachuted”, injected intravenously, smoked or insufflated [11,12]. Extraction methods are well described online, and five inhalers (containing 250 mg propylhexedrine each) provide one gram of “stove top speed at a fraction of the cost of street speed” [7]. Reported doses ingested to achieve psychoactive effects by online users range from 125 mg to 1250 mg [10,13]. Most devastating outcomes involve intravenous delivery, with one case report depicting an inadvertent intra-arterial injection leading to subsequent ischemic injuries [14]. Although relatively few case reports describe smoking extracted propylhexedrine, a case of “crack lung” was described in the setting of long-term smoking [11]. Parachuting (also called “bombing” or “dropping”) propylhexedrine, as in our case, has been described on user forums, as well as through various scientific reports at conferences [15,16]. Through this method, the user experiences a sustained-release effect as the “parachute” dissolves or unravels while avoiding the unpleasant taste of the chemical. This however increases the risk of overdose as well as the risk of bowel obstruction or perforation [15].

Propylhexedrine is generally regarded as a stimulant of last resort [1]. One online user claimed that “propylhexedrine is for stim hipsters and people whose meth dealer is out of town until next week” [10]. Propylhexedrine abuse leads to disinhibition, euphoria, analysis enhancement, anxiety, compulsive redosing, ego inflation, focus enhancement, increased libido, increased music appreciation, memory enhancement, motivation enhancement, thought acceleration, thought organization, and wakefulness [10, 17]. Some users report a pronounced “comedown” from this agent upon prolonged use which is marked by anxiety, appetite suppression, cognitive fatigue, irritability, depression, motivation suppression, thought deceleration and insomnia.

Adverse effects

Use of propylhexedrine can lead to increase in heart rate and blood pressure which can last for prolonged periods. Other physical symptoms associated with propylhexedrine abuse include: bronchodilation, dehydration, urinary frequency, hyperthermia, perspiration, bruxism, and temporary erectile dysfunction (Table 1). With these higher doses in the context of abuse, the emergence of arrhythmias has also been noted. One publication presented 15 cases of intravenous propylhexedrine-related deaths, majority being associated with anatomical indications of right ventricular hypertrophy and pulmonary hypertension at autopsy [6]. As a vasoconstrictor, it can disrupt the body’s natural cooling mechanisms and increase the risk of overheating and dehydration. Such toxicities can result from both single and chronic use. Intestinal impaction and aspiration are possible through parachuting. Stimulant-induced psychosis has also been reported, marked by paranoia, hallucinations, delusions and agitation as seen in our case. Upon abrupt cessation of use, online users report generalized discomfort, dysphoria and headache, however duration of such symptoms is unknown.

**Table 1:** Adverse effects of propylhexedrine intoxication gleaned from user forums

System		Reported symptom
Constitutional		Generalized discomfort
Eyes, Ears, Mouth, Throat		Tinnitus, vision changes
Cardiovascular		Chest discomfort, tachycardia, dizziness / postural presyncope, hypertension & hypertensive emergency
Respiratory		Shortness of breath
Gastrointestinal		Nausea, emesis, dysphagia, abdominal pain and appetite suppression
Neurologic		Tingling sensations, numbness, analgesia, tremor, headache, seizure
Psychiatric		Euphoria, tactile hallucinations, alterations of visual perception and visual hallucinations, alterations of auditory perception, decreased attention, appetite suppression, increased socialization, increased confidence, increased motivation, insomnia

Management

There is no specific reversal agent in cases of acute intoxication, and management is primarily symptomatic and supportive. Major issues that physicians may have to manage in the context of intoxication include severe agitation, tachycardia, hypertension, myocardial infarction, hyperthermia, stroke, bowel obstruction, pulmonary hypertension and seizures. Long-term use can also lead to lung damage, arrhythmias and cardiac structural damage. No guidelines exist for maintenance of long-term abstinence.

Discussion

Early literature underestimated the abuse potential of propylhexedrine. Smith et al.’s 1988 paper argued that despite the increased prevalence of cocaine and methamphetamine abuse at that time, there were no risk indicators of propylhexedrine abuse, and that it did not pose a significant public health concern at that time [1]. As seen in our paper, there is plenty of evidence from literature and user forums to suggest there are psychoactive effects comparable to amphetamines and inferior to that of methamphetamines with doses in the 100-300 mg range. Our patient depicted in our report experienced a transient state of psychotic delirium prompting admission, and this very quickly resolved. Among many others, the most concerning medical sequelae is the cardiovascular strain and the risk of heart damage from each exposure. Although propylhexedrine does not appear to be a one’s primary substance of abuse, it is a readily available alternative for those without access to their stimulant of choice. Given its chemical structure, use can evade detection on standard toxicology screens. The dissemination of abuse strategies and online forum discussions concerning psychoactive effects have the potential to increase the popularity of this substance even further and hence the incidence of toxicity cases and mortality.

From a legislative standpoint, Fernandez and Francis note that propylhexedrine is not chemically classified as an amphetamine and was therefore not included in the Combat Methamphetamine Epidemic Act of 2005 which increased scrutiny of products containing ephedrine, pseudoephedrine and norpseudoephedrine. Propylhexedrine therefore remains easily available for purchase over-the-counter or online with no restriction on amount. The World Health Organization Expert Committee on Drug Dependence first reviewed scheduling propylhexedrine in 1985, followed by reviews in 1985, 1989 and 1991 [7]. As of August 2020, it remains unscheduled.

## Conclusions

Propylhexedrine was intended to replace the highly stigmatized amphetamines in nasal decongestant inhalers with reassurance of a low abuse potential. Products containing propylhexedrine are currently available over the counter to anyone with the means to purchase it for a very low price. As a structural amphetamine analog, it is attractive to individuals seeking psychoactive effects to abuse it at above label doses for stimulant-like effects. At these higher doses used in abuse, significant cardiac side effects can result. In this paper we raise awareness of the potential for abuse of this readily accessible amphetamine analogue and suggest possible regulatory control over the sale of containing products.
